# Twin-to-twin transfusion syndrome and neonatal acute kidney injury after selective fetoscopic laser photocoagulation

**DOI:** 10.1007/s00467-026-07232-7

**Published:** 2026-03-17

**Authors:** Evelyn Obregon, Stefanie Riddle, Liu Chunyan, Shelley Ehrlich, Braxton Forde, Meredith P. Schuh, Stuart L. Goldstein, Cara L. Slagle

**Affiliations:** 1 Division of Neonatology, Perinatal Institute, Cincinnati Children’s Hospital Medical Center, 3333 Burnet Ave, Cincinnati, OH 45206 USA; 2 Fetal Care Center, Cincinnati Children’s Hospital Medical Center, Cincinnati, OH USA; 3 Division of Biostatistics and Epidemiology, Cincinnati Children’s Hospital Medical Center, Cincinnati, OH USA; 4 Division of Maternal-Fetal Medicine, Department of Obstetrics and Gynecology, University of Cincinnati College of Medicine, Cincinnati, USA; 5 Division of Nephrology & Hypertension, Cincinnati Children’s Hospital Medical Center, Cincinnati, OH USA; 6 Department of Pediatrics, University of Cincinnati College of Medicine, Cincinnati, OH USA; 7 Division of Neonatal-Perinatal Medicine, Department of Pediatrics, Indiana University School of Medicine, Indianapolis, IN USA

**Keywords:** Neonatal AKI, Twin-to-twin transfusion syndrome, Laser therapy

## Abstract

**Background:**

Selective Fetoscopic Laser Photocoagulation (SFLP) is used to treat Twin-Twin Transfusion Syndrome (TTTS), affecting 10–15% of monochorionic twin pregnancies. Historical data have reported neonatal acute kidney injury (AKI) as high as 50% following TTTS; however, the incidence of AKI following SFLP remains poorly characterized.

**Methods:**

We conducted a single-center retrospective study of monochorionic diamniotic twins who underwent SFLP for TTTS between 2010 and 2023. The primary outcome was the development of AKI during the first two weeks of life, defined by the Neonatal Modified KDIGO criteria. Mixed-effects logistic regression was performed to identify associations with AKI.

**Results:**

Among 103 infants included, 15 (14.6%) developed AKI. Twins with AKI were born at earlier gestational age (26 weeks vs. 30 weeks, *p* < 0.001) and had lower birth weights (923 vs. 1320 g, *p* = 0.01). Adjusted analysis demonstrated longer latency to delivery (OR 0.69, 95% CI 0.54–0.88) and greater total number of arteriovenous (AV) anastomoses (OR 0.78, 95% CI 0.64–0.94) were associated with decreased odds of AKI.

**Conclusion:**

Our postnatal incidence of AKI following SFLP for TTTS is less common than previously described. Our findings suggest that a more extensive AV anastomotic network and/or longer latency to delivery may allow for improved hemodynamic balance and kidney maturation.

**Graphical Abstract:**

**Figure:**
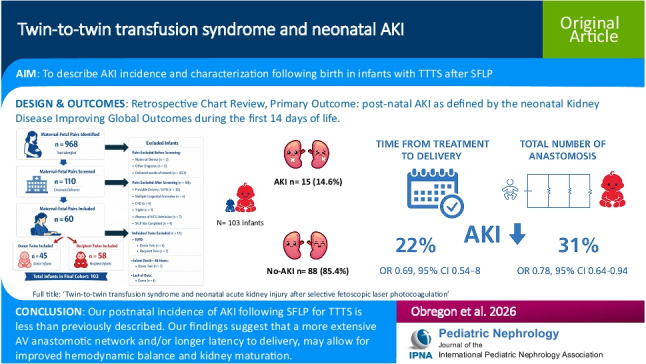
A higher resolution version of the Graphical abstract is available as Supplementary information

**Supplementary information:**

The online version contains supplementary material available at 10.1007/s00467-026-07232-7.

## Introduction

Twin-Twin Transfusion Syndrome (TTTS) is a serious complication that occurs in 10–15% of monochorionic twin pregnancies, or about 1 in 2000 pregnancies. TTTS results from a chronic imbalance of blood flow between monochorionic twins, characterized by unidirectional transfusion from the donor twin to the recipient twin through shared placental vascular anastomoses [1]. TTTS is detected by prenatal ultrasound and is characterized by the presence of oligohydramnios in the donor due to hypovolemia, kidney hypoperfusion, and oliguria, with polyhydramnios in the recipient twin due to hypervolemia and polyuria. Clinical management is determined by Quintero staging [2], which is based upon measurement of amniotic fluid volumes, bladder filling, Doppler abnormalities, presence of hydrops, and fetal demise, all ordered in five stages of severity [2]. The optimal treatment of TTTS is coagulation of the vascular anastomoses with Selective Fetoscopic Laser Photocoagulation (SFLP). Other treatment options, such as serial amnioreductions, can be used to reduce the risk of preterm birth, but do not treat the underlying pathophysiology. SFLP of these shared placental vascular connections can be offered as early as stage 1 TTTS and is considered the standard of care for treatment for stage 2 and above [3–6]. Previous studies before widespread use of SFLP reported a neonatal acute kidney injury (AKI) incidence following birth of approximately 50% [7] attributed to histological kidney changes such as hypovascularization and microangiopathy [8, 9]. Theoretically, the use of SFLP should improve the postoperative donor kidney function by stabilizing blood pressure and volume. However, the incidence of postnatal AKI in twins with TTTS who are treated with SFLP is not well known.

## Methods

This was a single-center retrospective study of monochorionic diamniotic twins who underwent SFLP for TTTS at a high-volume fetal care center between 2010 and 2023 and were admitted to local level III and IV Neonatal Intensive Care Units (NICU). This study was approved by the *Cincinnati Children’s Hospital Institutional Review Board* (IRB) (#2023–0075), with a waiver of informed consent given the retrospective nature of the study. The fetal care center database was used to identify patients and screened for delivery and admission to our institution. Single surviving twins were excluded during the review. Data collection was completed through chart review of maternal and infant medical records from participating local institutions. Separate institutional review board approval was obtained at each site as required. Maternal exclusion criteria included the following: patients delivered outside of network, multiple congenital anomalies, congenital heart disease, and/or higher order gestation. In cases where the mother met exclusion criteria, all corresponding twin infants were also excluded from the analysis. Individual twins were excluded for in utero fetal demise, absence of NICU admission and/or neonatal demise within 48 h. Data collected included maternal and infant basic demographics, medical course including surgical procedures, imaging results, medications and laboratory values. All information was transcribed directly into a REDCap database [10]. Strengthening The Reporting of OBservational studies in Epidemiology (STROBE) guidelines were followed [11].

### Outcome

The primary outcome was the development of AKI based on the Neonatal Modified Kidney Diseases Improving Global Outcomes (KDIGO) criteria (Appendix 1) during the first 2 weeks of life [12]. For this study, both the serum creatinine (sCr) criteria and/or the urine output (UOP) criteria definitions were used. Two physician adjudicators were used to determine the diagnosis of AKI to ensure reproducibility. In the event of a discrepancy, the chart was reviewed as a group and discussed until a conclusion could be determined.

### Statistical analysis

Descriptive statistics were used to summarize maternal and infant characteristics. Maternal variables were analyzed by comparing pregnancies with twins unaffected by AKI to those in which at least one twin was diagnosed with AKI. Chi-square or Fisher’s exact tests were used as appropriate for categorical variables and the Wilcoxon Rank Sum test or Kruskal–Wallis test for continuous variables given their non-normal distribution. The infant variables were analyzed across AKI groups using generalized mixed-effect models, with AKI as the sole fixed variable and the mother considered a random effect.

Mixed-effects logistic regression analyses were performed to identify the factors associated with AKI. Covariates included in the model were determined by *p*-values ≤ 0.05 in the bivariate analysis and clinical relevance, based on prior studies [13]. The correlation coefficients among the candidate variables were examined to avoid collinearity. If a high correlation was observed, the variable with the lowest *p*-value in the bivariate analyses with the outcome was chosen for inclusion in the multivariable model. Negative twice the residual log pseudo-likelihood was used to compare models. Parsimonious model was preferred when choosing the final model. SAS statistical software version 9.4 was used for all analyses. All tests were two-sided, and the significance level was set at a *p*-value < 0.05.

## Results

**Fig. 1:**
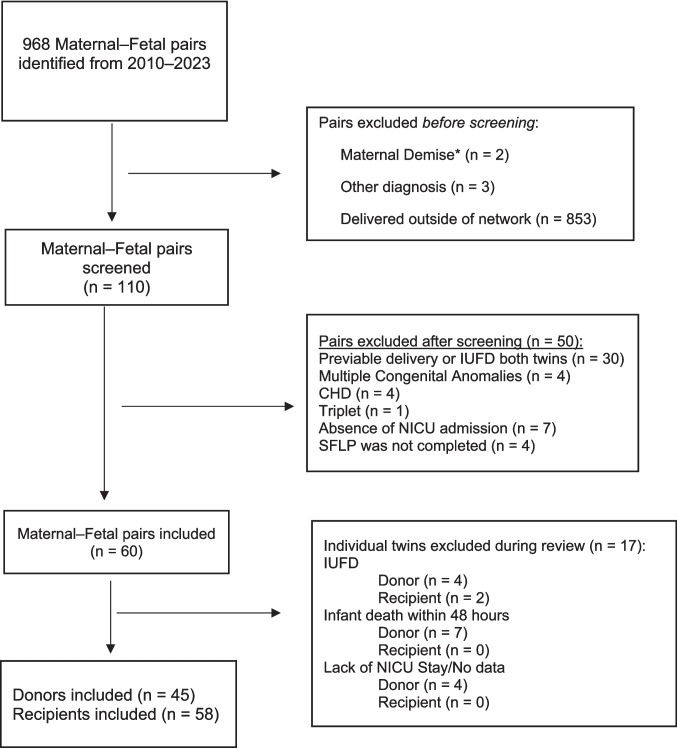
STROBE flow diagram. *Maternal demise not related to the procedure

During the study period, 60/968 (6%) of maternal–fetal pairs met inclusion criteria, resulting in a final cohort of 103 infants (45 donors, 58 recipients). Some individual twins were excluded due to in utero fetal demise, infant death within 48 h, or lack of NICU admission (Fig. 1).

There were no statistically significant differences in maternal demographics, comorbidities, obstetric complications, or corticosteroid administration between pregnancies in which at least one twin experienced AKI, compared to those in which neither twin was affected. However, AKI was more common following pregnancies in which indomethacin was used for tocolysis (53.8% vs. 23.4%, *p* = 0.04), but no significant differences were observed with other nephrotoxic agents [14] (Table 1).

**Table 1:** Maternal characteristics

Variable	Twins without AKI (*N* = 47)	At least one twin with AKI (*N* = 13)	Overall (*N* = 60)	*p*-value*
Demographics				
Maternal age in years, mean (SD)	30.4 (5.39)	29.3 (5.82)	30.2 (5.46)	0.47
Maternal race, *n* (%)				
Black or African American	3 (6.4%)	3 (25.0%)	6 (10.2%)	0.10
Hispanic	3 (6.4%)	-	3 (5.1%)	
Other	1 (2.1%)	-	1 (1.7%)	
White	40 (85.1%)	8 (66.7%)	48 (81.4%)	
Multiracial	-	1 (8.3%)	1 (1.7%)	
Maternal comorbidities, *n* (%)				
Chronic hypertension	7 (14.9%)	1 (7.7%)	8 (13.3%)	0.67
Obesity	4 (8.5%)	3 (23.1%)	7 (11.7%)	0.16
Hypothyroidism/hyperthyroidism	3 (6.4%)	-	3 (5.0%)	> 0.99
Non gestational diabetes	1 (2.1%)	1 (7.7%)	2 (3.3%)	0.39
Maternal obstetric complications, *n* (%)				
Gestational diabetes	6 (12.8%)	1 (7.7%)	7 (11.7%)	> 0.99
Pre-eclampsia/eclampsia/HELLP	3 (6.4%)	1 (7.7%)	4 (6.7%)	> 0.99
Short cervix	14 (29.8%)	6 (46.2%)	20 (33.3%)	0.32
Chorion amnion separation (CAS)	13 (27.7%)	4 (30.8%)	17 (28.3%)	> 0.99
Premature rupture of membranes (PROM), *n* (%)	26 (55.3%)	5 (38.5%)	31 (51.7%)	0.28
GA at PROM (weeks), mean (SD)	28 (3.99)	25 (2.06)	27 (3.89)	0.13
Prolonged PROM, *n* (%)	22 (46.8%)	3 (23.1%)	25 (41.7%)	0.12
Chorioamnionitis, *n* (%)	9 (19.1%)	1 (7.7%)	10 (16.7%)	0.44
Tocolysis, *n* (%)				
Magnesium	20 (42.6%)	8 (61.5%)	28 (46.7%)	0.22
Indomethacin	11 (23.4%)	7 (53.8%)	18 (30.0%)	0.04
Nifedipine	37 (78.7%)	9 (69.2%)	46 (76.7%)	0.48
Maternal medications, *n* (%)				
Nephrotoxins	34 (75.6%)	7 (53.8%)	41 (70.7%)	0.17
Antenatal steroids	44 (95.7%)	12 (92.3%)	56 (94.9%)	0.53
Repeat course of steroids	16 (42.1%)	6 (54.5%)	22 (44.9%)	0.51
Antibiotics before delivery, *n* (%)				
Azithromycin and cefazolin	9 (20.5%)	3 (25.0%)	12 (21.4%)	0.84
Cefazolin	20 (45.5%)	6 (50.0%)	26 (46.4%)	
Other	15 (34.1%)	3 (25.0%)	18 (32.1%)	
Delivery				
Maternal creatinine, mean (SD)	0.53 (0.11)	0.59 (0.14)	0.54 (012)	0.36
Mode of delivery, *n* (%)				
C-section	38 (80.9%)	10 (76.9%)	48 (80.0%)	0.71
Vaginal	9 (19.1%)	3 (23.1%)	12 (20.0%)	
Indication for C-section, *n* (%)				
Previous C-section	5 (10.6%)	1 (7.7%)	6 (10.0%)	> 0.99
Malpresentation	9 (19.1%)	5 (38.5%)	14 (23.3%)	0.16
Preterm labor	21 (44.7%)	6 (46.2%)	27 (45.0%)	0.92
Chorioamnionitis	7 (14.9%)	-	7 (11.7%)	0.33
Non-reassuring fetal heart tracing	11 (23.4%)	3 (23.1%)	14 (23.3%)	> 0.99

^*^Wilcoxon rank sum test for continuous variables and Chi-square or Fisher’s exact test for categorical variables

In pregnancies where at least one twin was diagnosed with AKI, the mean gestational age (GA) at SFLP was 22 (SD ± 2.82) weeks with the majority classified as Quintero Stage III (69.2% vs. 56.4%, *p* = 0.95) (Table 2). These pregnancies had a lower total number of placental vascular anastomoses compared to those without AKI (12 vs. 22, *p* < 0.01) (Table 2). Both donor-to-recipient (D-R) anastomoses (5 vs. 9, *p* = 0.03) and recipient-to-donor (R-D) anastomoses (6 vs. 12, *p* = 0.02) were fewer. Additionally, latency to delivery (i.e., interval between SFLP and birth) was significantly shorter in pregnancies affected by AKI (4 weeks vs. 9 weeks, *p* < 0.01) (Table 2). Placental share among both groups was comparable for donors and recipients.

**Table 2:** SFLP characteristics

Variable	Twins without AKI (*N* = 47)	At least one twin with AKI (*N* = 13)	Overall (N = 60)	*p*-value*
Gestational age at TTTS diagnosis (weeks), mean (SD)	20 (2.87)	21 (3.30)	20 (2.96)	0.41
Quintero stage at diagnosis, *n* (%)				
I	10 (21.3%)	4 (30.8%)	14 (23.3%)	0.86
II	10 (21.3%)	2 (15.4%)	12 (20.0%)	
III	24 (51.1%)	6 (46.2%)	30 (50.0%)	
IV	3 (6.4%)	1 (7.7%)	4 (6.7%)	
Gestational age at SFLP (weeks), mean (SD)	21 (2.85)	22 (2.82)	21 (2.87)	0.15
Quintero stage at SFLP, *n* (%)				
I	5 (10.6%)	1 (7.7%)	6 (10.0%)	0.95
II	12 (25.5%)	2 (15.4%)	14 (23.3%)	
III	27 (57.4%)	9 (69.2%)	36 (60.0%)	
IV	3 (6.4%)	1 (7.7%)	4 (6.7%)	
Net amount of fluid removed (ml), mean (SD)	1671.32 (1131.46)	1775.54 (792.43)	1693.90 (1061.94)	0.53
Placental share donor (%), mean (SD)	33.21 (14.21)	35.00 (15.63)	33.57 (14.36)	0.75
Placental share recipient (%), mean (SD)	66.79 (14.21)	65.00 (15.63)	66.43 (14.36)	0.75
Total number of anastomoses	22 (12.05)	12.3 (4.87)	20 (11.63)	< 0.01
Anastomoses donor-to-recipient, mean (SD)	9.2 (7.09)	5 (3.32)	8.4 (6.68)	0.03
Anastomoses recipient-to-donor, mean (SD)	12.2 (7.99)	6.5 (3.62)	11 (7.68)	0.02
Intraamniotic antibiotics				
Clindamycin	8 (17.0%)	2 (15.4%)	10 (16.7%)	> 0.99
Nafcillin	39 (83.0%)	11 (84.6%)	50 (83.3%)	
Latency to delivery (weeks), mean (SD)	9 (4.28)	4 (3.45)	8 (4.50)	** < 0.01**

^*^Wilcoxon rank sum test for continuous variables and Chi-square or Fisher’s exact test for categorical variables

Among 103 infants, 15 (14.6%) were diagnosed with neonatal AKI in the first 2 weeks of life. Twins with AKI were born at earlier GA (26 weeks vs. 30 weeks, *p* < 0.01) and had lower birth weights (923 vs. 1320 g, *p* = 0.02) compared to those without AKI. Infants with AKI had a higher need for vasopressor use in their first week of life (26.7% vs. 10.2%, *p* = 0.09), increased incidence of respiratory distress (100% vs. 65.9%, *p* < 0.01), and required more days of mechanical ventilation (15 days vs. 9 days, *p* = 0.03). Twin status (i.e., donor vs. recipient) was not different between twins with and without AKI (Table 3). Other comorbidities, including grade 3 or 4 intraventricular hemorrhage, cardiovascular dysfunction, pulmonary hypertension, persistent ductus arteriosus, necrotizing enterocolitis, and exposure to nephrotoxic medications [14] were similar between both groups.

**Table 3:** Prenatal and postnatal infant characteristics

Variable	Non-AKI (*N* = 88)	Twin with AKI (*N* = 15)	Overall (*N* = 103)	p*
Prenatal characteristics				
Selective fetal growth restriction, *n* (%)	27 (31.0%)	8 (53.3%)	35 (34.3%)	0.11
Discordant (%), mean (SD)	28.05 (10.02)	27.63 (4.75)	27.93 (8.84)	0.54
TAPS (twin anemia polycythemia syndrome), *n* (%)	12 (13.8%)	4 (26.7%)	16 (15.7%)	0.32
Abnormal initial umbilical artery Doppler, *n* (%)	26 (30.2%)	3 (20.0%)	29 (28.7%)	0.43
Normal umbilical artery Doppler after SFLP, *n* (%)	61 (75.3%)	12 (92.3%)	73 (77.7%)	0.25
Abnormal UA Doppler before delivery	18 (20.9%)	2 (13.3%)	20 (19.8%)	0.52
Deep vertical pocket (DVP) before SFLP (cm), mean (SD)	6.66 (5.13)	4.58 (4.75)	6.35 (5.11)	0.15
DVP after SFLP (cm), mean (SD)	5.22 (2.67)	4.31 (3.46)	5.09 (2.78)	0.28
DVP before delivery (cm), mean (SD)	4.44 (2.37)	4.22 (3.30)	4.41 (2.50)	0.89
Cardiac disfunction on fetal echo at diagnosis, *n* (%)	8 (12.1%)	-	8 (10.3%)	0.98
Cardiac disfunction after SFLP, *n* (%)	8 (10.7%)	3 (25.0%)	11 (12.6%)	0.18
Cincinnati staging				
A	7 (8.0%)	3 (20.0%)	10 (9.7%)	0.21
B	9 (10.2%)	-	9 (8.7%)	
C	35 (39.8%)	4 (26.7%)	39 (37.9%)	
Not applicable	37 (42.0%)	8 (53.3%)	45 (43.7%)	
Infant characteristics				
Gestational age at birth (weeks), mean (SD)	29.70 (2.99)	26.38 (2.50)	28.98 (3.19)	< 0.01
Birth weight (grams), mean (SD)	1320.40 (479.40)	923.27 (384.03)	1262.56 (485.89)	0.02
Twin status, *n* (%)				
Donor	36 (40.9%)	9 (60.0%)	45 (43.7%)	0.18
Recipient	52 (59.1%)	6 (40.0%)	58 (56.3%)	
Fetal distress, *n* (%)	30 (34.5%)	6 (40.0%)	36 (35.3%)	0.68
Number of serum creatinine, mean (SD)	5.61 (2.74)	9.87 (3.68)	6.23 (3.24)	< 0.01
APGAR 1 min, mean (SD)	5.57 (2.54)	5.00 (3.05)	5.49 (2.61)	0.48
APGAR 5 min, mean (SD)	7.66 (1.91)	6.93 (2.55)	7.55 (2.02)	0.50
Comorbidities				
Need for vasopressors, first week of life, *n* (%)	9 (10.2%)	4 (26.7%)	13 (12.6%)	0.09
Respiratory distress in first week, *n* (%)	58 (65.9%)	15 (100%)	73 (70.9%)	< 0.01
Invasive respiratory support, *n* (%)	39 (50.0%)	11 (73.3%)	50 (53.8%)	0.15
Mechanical ventilation (days), mean (SD)	9.17 (28.82)	14.93 (20.36)	10.03 (27.72)	0.03
Air leak syndrome, *n* (%)	2 (2.3%)	-	2 (1.9%)	0.98
Sepsis, *n* (%)	19 (21.6%)	5 (33.3%)	24 (23.3%)	0.46
Intra ventricular hemorrhage G3-G4, *n* (%)	8 (9.1%)	2 (13.3%)	10 (9.7%)	0.57
Other neurological injuries (PVL/ischemic etc.), *n* (%)	13 (14.8%)	5 (33.3%)	18 (17.5%)	0.13
Left ventricular dysfunction, *n* (%)	4 (19.0%)	-	4 (13.3%)	0.29
Pulmonary hypertension, *n* (%)	1 (25.0%)	2 (66.7%)	3 (42.9%)	0.69
Persistent ductus arteriosus (PDA), *n* (%)	15 (71.4%)	6 (60.0%)	21 (67.7%)	0.52
Necrotizing enterocolitis, *n* (%)	9 (10.2%)	2 (13.3%)	11 (10.7%)	0.72
Intestinal perforation, *n* (%)	1 (1.1%)	1 (6.7%)	2 (1.9%)	0.22
Nephrotoxic medications^a^ (doses), mean (SD)	2.66 (3.30)	3.73 (2.40)	2.86 (3.17)	0.46
Aminoglycosides (doses), mean (SD)	1.54 (1.24)	1.86 (0.77)	1.60 (1.17)	0.54

*p-values were from mixed effect model where mom was used as random effect for infant-level data. Fisher’s exact test was used for categorical variables when non-convergence occurred for mixed model. The Wilcoxon rank sum test for mom-level continuous variables. When *N* is small, no test was done

^a ^common nephrotoxic medications included

Among the 15 infants diagnosed with AKI, 8 met diagnostic criteria based on sCr and 3 based on UOP, while 4 infants met criteria based on both parameters (Appendix 2). Infants who met both sCr and UOP criteria had higher stages of AKI compared to those who met criteria based on either parameter alone. There were no differences between the groups in terms of AKI duration, exposure to nephrotoxic medications [14], or twin status (Table 4). Interestingly, only 5 of the 15 infants with AKI had the condition documented in their problem list, and nephrology was consulted in just one of these cases.

**Table 4:** AKI characteristics by diagnosis criteria

Variable	Creatinine OR UOP (*N* = 11)	Creatinine AND UOP (*N* = 4)	Total (*N* = 15)	*p*-value*
AKI stage, *n* (%)				
I	7 (63.6%)		7 (46.7%)	0.06
II	3 (27.3%)	2 (50.0%)	5 (33.3%)	
III	1 (9.1%)	2 (50.0%)	3 (20.0%)	
Duration of AKI (days), mean (SD)	3.36 (3.59)	6.75 (4.92)	4.27 (4.10)	0.14
Nephrotoxic medications (doses), mean (SD)	3.45 (2.70)	4.50 (1.29)	3.73 (2.40)	0.26
Twin status, *n* (%)				
Donor	7 (63.6%)	2 (50.0%)	9 (60.0%)	> 0.99
Recipient	4 (36.4%)	2 (50.0%)	6 (40.0%)	
Kidney ultrasound, *n* (%)				
Abnormal	-	1 (25.0%)	1 (6.7%)	0.48
Not done	10 (90.9%)	3 (75.0%)	13 (86.7%)	
Normal	1 (9.1%)	-	1 (6.7%)	
AKI defined in problem list, *n* (%)	3 (27.3%)	2 (50.0%)	5 (33%)	0.56
Nephrology consult, *n* (%)	-	1 (25.0%)	1 (7%)	0.27

^*^p-values were from the Kruskal–Wallis test for continuous variables and Fisher’s exact test for categorical variables

In unadjusted analysis, birth weight and GA were associated with AKI, but vasopressor need in the first week of life did not affect the outcome. Since gestational age (GA) and birth weight were highly correlated with latency to delivery, the latter was kept to minimize the number of covariates due to limited sample size. Twin status, latency to delivery, deep vertical pocket before SFLP and total number of anastomoses were examined in the mixed model while controlling for hypotension in the first week and gestational age at birth.

Final covariates included in the model were latency to delivery and total number of anastomoses. Adjusted analysis showed that the main predictors of AKI were latency to delivery (aOR 0.69, 95% CI 0.54–0.88, *p* < 0.01) and total number of arteriovenous (AV) anastomoses (aOR 0.78, 95% CI 0.64–0.94, *p* = 0.01).

## Discussion

TTTS-related hemodynamic stress and prematurity create a unique high-risk environment for subsequent neonatal AKI. Our study found a lower incidence of AKI (14.6%) in infants affected by TTTS after SFLP compared to historical rates reported in the general NICU population [12]. In our cohort, both donor and recipient twins were equally affected, and despite increased awareness, reporting of AKI by providers remains low. Additionally, each additional week of gestation after SFLP was associated with a 31% reduced risk of AKI and one more anastomosis with a 22% reduction of AKI.

Earlier studies of AKI among infants affected by TTTS before SFLP became common practice reported the incidence of AKI to be around 50%, with equal incidences among donor and recipient twins [7]. Additionally, one study demonstrated elevated biomarkers of kidney dysfunction in infants with TTTS compared to dichorionic-diamniotic twins, further highlighting the renal vulnerability in this population [15]. More recent studies conducted after the introduction of SFLP have shown significant reductions in AKI incidence among TTTS survivors [16]. However, these studies often lacked standardized definitions of AKI or employed high serum creatinine thresholds, which may have led to underestimation of AKI rates and hindered meaningful comparisons [17]. Notably, Melhem et al. [18] utilized the KDIGO criteria and reported an AKI incidence of 50%, specifically grade 2 and 3 AKI, even after SFLP, highlighting the persistent risk and the importance of using consistent diagnostic criteria (Appendix 3).

Prior to SFLP, the donor twin is often hypovolemic with reduced kidney perfusion and oliguria, and the recipient is hypervolemic which may cause kidney congestion. The sudden cessation of intertwin transfusion following laser therapy can lead to rapid hemodynamic shifts, which may further compromise kidney blood flow [19]. Given that SFLP is able to be completed in > 98% of fetoscopic surgeries for twins, we routinely use the sequential technique for all SFLPs. Intraoperatively, the surgeon evaluates the number of vascular anastomoses and estimates the placental share prior to ablation. The sequential technique involves the initial ablation of arteriovenous (AV) anastomoses with unidirectional blood flow from donor to the recipient, followed by laser occlusion of AV anastomoses from recipient to donor [19]. By staging the interruption of blood flow, the sequential method may offer a more targeted and physiologically adaptive strategy, allowing a brief period to rebalance the relative hypo and hypervolemia in each twin. This may mitigate the risk of AKI in these vulnerable preterm infants [20]. However, direct comparative data on AKI incidence between laser techniques is lacking in the literature.

While AV anastomoses are typically implicated in the pathogenesis of TTTS, having additional AV anastomoses may paradoxically reduce the risk of AKI. Multiple AV connections in both directions can help distribute blood flow more evenly between the twins, thereby reducing net volume imbalance. This configuration may also lower the pressure gradient across individual vessels, reducing the likelihood of abrupt hemodynamic shifts that compromise kidney perfusion [21]. Furthermore, a more complex vascular network even with more AV anastomoses may mitigate the severity of TTTS by allowing better hemodynamic compensation.

There is limited research linking longer latency between SFLP and birth and the risk of neonatal AKI. However, several physiological considerations support this possibility. Beyond the intuitive benefit that prolonged gestation allows for continuing kidney maturation and reduced need for intensive neonatal interventions, extended latency may also facilitate the gradual restoration of kidney perfusion [22]. This interval provides time for the fetal cardiovascular system to adapt and stabilize following SFLP. Additionally, it may allow the kidneys to recover from the chronic hemodynamic disturbances present prior to intervention due to TTTS and thus mitigate the risk of postnatal AKI. Beck et al. [23] reported normal kidney function at 3 years of age in 18 surviving twins after SFLP. They concluded that early laser treatment for TTTS (median 20.7 weeks) prevents long-term kidney impairment in surviving twins. However, the precedence of AKI in the neonatal period was unclear.

Our study has several notable limitations. This is a single-center and retrospective analysis with a relatively small cohort and lack of control group, limiting generalizability. Second, many maternal–fetal dyads who underwent SFLP at our institution subsequently returned to their referring centers for delivery, resulting in incomplete data capture. As one of the largest quaternary referral centers, our population may also be skewed toward more complex cases, since patients with complicated post-procedural courses are more likely to remain in our care. Lastly, as impaired kidney function occurs in utero, post-natal AKI may not reflect future long-term kidney outcomes. Despite these limitations, the strengths of this study include the use of standardized contemporary diagnostic criteria for AKI, ensuring consistency with current literature and clinical practices. Additionally, our cohort reflects current procedural and perinatal management practices, enhancing the relevance of our findings.

## Conclusion

In conclusion, our findings suggest that the incidence of neonatal AKI following SFLP has decreased. A complex network of AV anastomoses may influence the risk of AKI by modulating blood flow distribution and hence the importance of mitigating abrupt circulatory shifts and preserving kidney perfusion. Our results could support further research that apply techniques such as sequential SFLP to re-balance the blood volume distribution and thus improve kidney perfusion sooner after surgery. Furthermore, this technique, when followed by a longer latency to delivery, not only allows for kidney maturation, but provides a critical time for the fetus to recover from chronic circulatory insults.

## Supplementary information

Below is the link to the electronic supplementary material.

ESM 1 (DOCX 21.2 KB)

ESM 2 (DOCX 21.6 KB)

Graphical abstract (PPTX 1.15 MB)

## Data Availability

The datasets generated during and/or analyzed during the current study are available from the corresponding author on reasonable request.
