# Supplementary material for: Twin-to-twin transfusion syndrome and neonatal acute kidney injury after selective fetoscopic laser photocoagulation
Source: Pediatr Nephrol. 2026 Mar 17;41(8):2647–55. doi: 10.1007/s00467-026-07232-7 (PMC13337727; doi:10.1007/s00467-026-07232-7)
Supplement: Supplementary file 1 — (DOCX 21.2 KB) [file 467_2026_7232_MOESM1_ESM.docx]

Appendix 1. Neonatal Modified Kidney Diseases Improving Global Outcomes (KDIGO) criteria

| **Stage** | **Serum creatinine** | **UOP over 24 hours** |
| --- | --- | --- |
| **0** | No change in serum creatinine *or* rise < 0·3 mg/dL | > 1 mL/kg/hour |
| **1** | SCr rise ≥ 0·3 mg/dL within 48 hours *or*  SCr rise ≥1·5–1·9 × reference SCr* within 7 days | >0·5 and ≤ 1 mL/kg/hour |
| **2** | SCr rise ≥ 2 to 2·9 × reference SCr* | >0·3 and ≤0·5 mL/kg/hour |
| **3** | SCr rise ≥ 3 × reference SCr * *or*  SCr ≥ 2·5 mg/dL** *or*  Receipt of dialysis | ≤ 0·3 mL/kg/hour |

*Reference SCr is the lowest prior SCr measurement
** this is lower than the original KDIGO definition as a SCr of 2.5 mg/dl in neonates suggests a GFR < 10 ml/min/1.73 m^2^
*SCr* = serum creatinine, *UOP* = urine output

Appendix 2. AKI characteristics by diagnosis criteria (expanded)

| **Variable** | **UOP (N = 3)** | **Creatinine (N = 8)** | **Both (N = 4)** | **Total (N = 15)** | **P-value*** |
| --- | --- | --- | --- | --- | --- |
| **AKI diagnosis** | | | | | |
| **AKI stage** |  |  |  |  | 0.0996 |
| I | 3 (100%) | 4 (50.0%) |  | 7 (46.7%) |  |
| II |  | 3 (37.5%) | 2 (50.0%) | 5 (33.3%) |  |
| III |  | 1 (12.5%) | 2 (50.0%) | 3 (20.0%) |  |
| **Duration of AKI (days)** |  |  |  |  | 0.1572 |
| N | 3 | 8 | 4 | 15 |  |
| Min–Max | 1.0–2.0 | 1.0–12.0 | 2.0–11.0 | 1.0–12.0 |  |
| Mean (SD) | 1.33 (0.58) | 4.13 (3.98) | 6.75 (4.92) | 4.27 (4.10) |  |
| Median (Q1, Q3) | 1.0 (1.0, 2.0) | 3.0 (1.0, 6.0) | 7.0 (2.5, 11.0) | 2.0 (1.0, 8.0) |  |
| Missing | 0 | 0 | 0 | 0 |  |
| **Nephrotoxic medications (days)** |  |  |  |  | 0.1532 |
| N | 3 | 8 | 4 | 15 |  |
| Min–Max | 2.0–8.0 | 1.0–8.0 | 3.0–6.0 | 1.0–8.0 |  |
| Mean (SD) | 5.33 (3.06) | 2.75 (2.38) | 4.50 (1.29) | 3.73 (2.40) |  |
| Median (Q1, Q3) | 6.0 (2.0, 8.0) | 2.0 (1.0, 3.5) | 4.5 (3.5, 5.5) | 3.0 (2.0, 6.0) |  |
| Missing | 0 | 0 | 0 | 0 |  |
| **Twin status** |  |  |  |  | 0.3958 |
| Donor | 3 (100%) | 4 (50.0%) | 2 (50.0%) | 9 (60.0%) |  |
| Recipient |  | 4 (50.0%) | 2 (50.0%) | 6 (40.0%) |  |
| **Kidney ultrasound** |  |  |  |  | 0.7333 |
| Abnormal |  |  | 1 (25.0%) | 1 (6.7%) |  |
| Not done | 3 (100%) | 7 (87.5%) | 3 (75.0%) | 13 (86.7%) |  |
| Normal |  | 1 (12.5%) |  | 1 (6.7%) |  |
| **AKI defined in problem list** |  |  |  |  | 0.7762 |
| No | 2 (66.7%) | 6 (75.0%) | 2 (50.0%) | 10 (66.7%) |  |
| Yes | 1 (33.3%) | 2 (25.0%) | 2 (50.0%) | 5 (33.3%) |  |
| **Nephrology consult** |  |  |  |  | 0.4667 |
| No | 3 (100%) | 8 (100%) | 3 (75.0%) | 14 (93.3%) |  |
| Yes |  |  | 1 (25.0%) | 1 (6.7%) |  |
| * P-values were from Kruskal–Wallis test for continuous variables and Fisher's exact test for categorical variables | | | | | |
