# Supplementary material for: Twin-to-twin transfusion syndrome and neonatal acute kidney injury after selective fetoscopic laser photocoagulation
Source: Pediatr Nephrol. 2026 Mar 17;41(8):2647–55. doi: 10.1007/s00467-026-07232-7 (PMC13337727; doi:10.1007/s00467-026-07232-7)
Supplement: Supplementary file 2 — (DOCX 21.6 KB) [file 467_2026_7232_MOESM2_ESM.docx]

| Year | Author | Country | Sample Size | Intervention | AKI definition | AKI incidence |
| --- | --- | --- | --- | --- | --- | --- |
| 2000 | Cincotta | Australia | 17 pregnancies affected by TTTS and 17 controls (29 infants with TTTS, 33 controls) | Serial amnioreductions only | UOP < 1 ml/kg/hr first 3 days | 48% TTTS groups  15% control |
| 2003 | Chiang | China | 19 pregnancies (33 live births) | Serial amnioreductions only | Cr > 1.5 mg/dl | 43% (8 donors, 1 recipient) |
| 2004 | **Introduction of laser therapy for TTTS** | | | | | |
| 2007 | Lenclen | France | 137 infants (36 amnioreduction, 101 laser) vs. 242 di-di twins | Amnioreduction and laser therapy | None | 20% Amnioreduction group  7% Laser group  1% di-di twins |
| 2011 | Mercanti | Canada | 51 pregnancies (51 donors, 51 recipients) | Serial amnioreductions | None | 22% Donors  20% Recipients |
| 2012 | Halvorsen | Sweden | 73 pregnancies (71 donors, 71 recipients) | Laser therapy | None | 10% Overall  12% Donors  8.7% Recipients |
| 2017 | Verbeek | Netherlands | 312 pregnancies (274 twins laser group, 38 twins non laser) | Laser and amnioreduction | Cr > 100 umol/l in the first week of life | 38% Non-laser group  7% Laser group |
| 2019 | Melhem | UK | 26 infants (10 in-utero intervention, 16 expectant) | Laser and amnioreduction | Neonatal KDIGO | 50% Laser group  56% Expectant/amnioreduction |

**Appendix 3.** Overview of published research on TTTS and neonatal AKI
