# Supplementary material for: Twin-to-twin transfusion syndrome and neonatal acute kidney injury after selective fetoscopic laser photocoagulation
Source: Pediatr Nephrol. 2026 Mar 17;41(8):2647–55. doi: 10.1007/s00467-026-07232-7 (PMC13337727; doi:10.1007/s00467-026-07232-7)
Supplement: Supplementary file 3 — Graphical abstract (PPTX 1.15 MB) [file 467_2026_7232_MOESM3_ESM.pptx]

## Slide 1
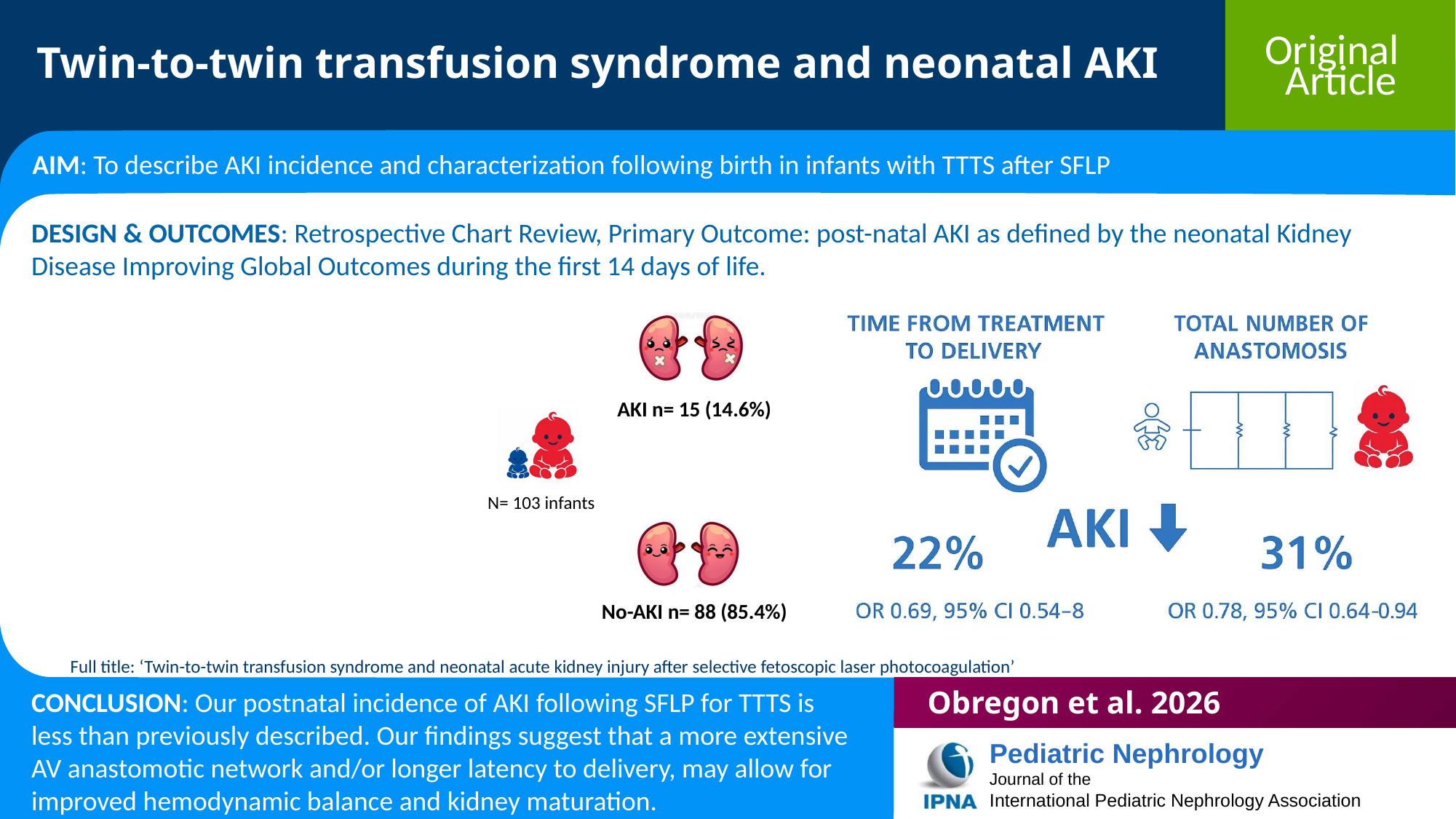

Twin-to-twin transfusion syndrome and neonatal AKI
AIM: To describe AKI incidence and characterization following birth in infants with TTTS after SFLP
DESIGN & OUTCOMES: Retrospective Chart Review, Primary Outcome: post-natal AKI as defined by the neonatal Kidney Disease Improving Global Outcomes during the first 14 days of life.
AKI n= 15 (14.6%)
N= 103 infants
No-AKI n= 88 (85.4%)
Full title: ‘Twin-to-twin transfusion syndrome and neonatal acute kidney injury after selective fetoscopic laser photocoagulation’
Obregon et al. 2026
CONCLUSION: Our postnatal incidence of AKI following SFLP for TTTS is less than previously described. Our findings suggest that a more extensive AV anastomotic network and/or longer latency to delivery, may allow for improved hemodynamic balance and kidney maturation.
